# Characterization of *In Vitro* Resistance to Linezolid in Mycobacterium abscessus

**DOI:** 10.1128/spectrum.02199-23

**Published:** 2023-07-17

**Authors:** Dereje A. Negatu, Wassihun Wedajo Aragaw, Véronique Dartois, Thomas Dick

**Affiliations:** a Center for Discovery and Innovation, Hackensack Meridian Health, Nutley, New Jersey, USA; b Center for Innovative Drug Development and Therapeutic Trials for Africa, Addis Ababa University, Addis Ababa, Ethiopia; c Department of Medical Sciences, Hackensack Meridian School of Medicine, Nutley, New Jersey, USA; d Department of Microbiology and Immunology, Georgetown University, Washington, D.C., USA; Johns Hopkins University School of Medicine

**Keywords:** MAB_4384, MmpL5-MmpS5, NTM, TetR, cross resistance, nontuberculous mycobacteria, oxazolidinones, rplC, rrl

## Abstract

Single-step selection of Mycobacterium abscessus mutants resistant to linezolid yielded high-level resistance at a low frequency that was associated with mutations in 23S rRNA or the ribosomal protein L3. Surprisingly, linezolid-resistant rRNA mutations conferred cross-resistance to several unrelated antibiotics. Low-level linezolid-resistant mutants were isolated at a higher frequency and were due to loss-of-function mutations in the transcriptional regulator MAB_4384, the repressor of the drug efflux pump MmpL5-MmpS5.

**IMPORTANCE** The protein synthesis inhibitor linezolid is used for the treatment of lung disease caused by Mycobacterium abscessus. However, many strains of the bacterium show poor susceptibility to the antibiotic. For most clinical isolates, resistance is not due to mutations in the target of the drug, the ribosome. The mechanism responsible for non-target-related, indirect linezolid resistance is unknown. Here, we analyzed the development of linezolid resistance in the M. abscessus reference strain *in vitro*. We found, as expected, resistance mutations in the ribosome. In addition, we identified mutations in a system that involves a drug pump, suggesting drug efflux as a mechanism of resistance to linezolid. This finding may inform the analysis of clinical resistance to linezolid. Surprisingly, a subset of linezolid-resistant ribosome mutations conferred cross-resistance to several structurally and mechanistically unrelated drugs, uncovering a novel multidrug resistance mechanism.

## INTRODUCTION

The prevalence of lung diseases caused by the rapidly growing nontuberculous mycobacterium species M. abscessus is increasing (1). Despite prolonged treatments, including an oral macrolide (azithromycin or clarithromycin) and various injectables, including amikacin, cefoxitin or imipenem, and tigecycline, cure rates are poor (2). Several additional oral drugs, including the oxazolidinone linezolid (LZD), are used for the treatment of M. abscessus lung disease (2). LZD is a synthetic antibiotic that interferes with protein synthesis by inhibiting the peptidyl transferase activity of the 23S rRNA in the 50S ribosomal subunit, resulting in ribosome stalling (3), similar to the molecular mechanism of the natural product chloramphenicol. A large fraction of clinical M. abscessus isolates show poor susceptibility to LZD (4, 5). Interestingly, most of these LZD-resistant isolates do not harbor mutations in the ribosome (5). Prior mRNA expression studies of candidate efflux pumps in clinical isolates suggested the involvement of efflux in indirect LZD resistance (5). However, functional (genetic) evidence for pump-related resistance mechanisms is lacking. Here, development of resistance against LZD was characterized *in vitro* using the type strain M. abscessus subsp. *abscessus* ATCC 19977 and a single-step selection approach. Two categories of resistance mechanisms, on- and off-target, were identified. Both resistance classes were profiled for cross-resistance to other oxazolidinones and to a panel of nonoxazolidinone anti-M. abscessus antibiotics.

## RESULTS AND DISCUSSION

### Low-frequency and high-level LZD resistance is due to mutations in 23S rRNA or ribosomal protein L3.

First, the development of high-level LZD resistance due to on-target mutations in the ribosome was recapitulated (6, 7). Two independent selection experiments on Middlebrook 7H10 agar containing a high drug concentration (400 μM; 16× the agar MIC of 25 μM) (Table 1) were carried out as described previously using M. abscessus ATCC 19977 (8). Candidate resistant colonies appeared after 5 to 10 days of incubation and were restreaked on agar containing the same 400 μM LZD concentration to confirm resistance. The frequency of spontaneous resistance (FOR) was 4 × 10^−9^/CFU. Seven resistant colonies (LZD_r1 to _r7, with LZD_r1 to _r5 forming colonies after 10 days and LZD_r6 and _r7 forming colonies after 5 days) (Table 1) were subjected to MIC determinations using the broth dilution method (Middlebrook 7H9), with the optical density at 600 nm (OD_600_) as a readout to generate dose-response curves. Growth curve measurements during the preparation of exponentially growing (drug-free) precultures for the growth inhibition experiments revealed that the subset of resistant strains taking 10 days to from visible colonies on agar (LZD_r1 to _r5 [Table 1]) showed an ~30% increase in generation time compared to the wild-type strain, suggesting that the resistance mutations in these strains are associated with a moderate growth defect. Table 1 displays growth inhibition activity of LZD against strains LZD_r1 to _r7, reported as the MIC_50_ and MIC_90_, i.e., the concentrations required to inhibit growth by 50% and 90% compared to drug-free control. As expected, all 7 strains showed a strong shift in their LZD MICs (Table 1). Whole-genome sequencing, confirmed by targeted Sanger sequencing, revealed the single-nucleotide polymorphism of a guanine-to-uracil change at position 2659 (G2659U; corresponding to G2447U with Escherichia coli numbering) in the peptidyl transferase center located in domain V of the 23S rRNA (*rrl*, MAB_r5052; strains LZD_r1 to _r5) (Table 1) and a single amino acid substitution (C142R) in the 50S ribosomal protein L3 (*rplC*, MAB_3820c; strains LZD_r6 and _r 7) (Table 1). Both polymorphisms were previously shown to confer high-level LZD resistance in other mycobacteria, likely by reducing binding of LZD to the peptidyl transferase center (7, 9, 10).

**TABLE 1:** Characterization of linezolid-resistant M. abscessus ATCC 19977 strains isolated in single-step selection experiments^*a*^

M. abscessus strain	Selection concn (μM LZD)	Expt no.^*b*^	FOR	LZD (μM)		SPR719 (μM)		Polymorphisms and affected genes				
				MIC_50_	MIC_90_	MIC_50_	MIC_90_	MAB gene	Mutation	Amino acid change	Gene name and function	Gene(s) with additional polymorphisms
WT	NA	NA	NA	3.5	15	0.25	1.5	WT	WT	WT		
LZD_r1	400	1	4 × 10^−9^	60	>100	ND	ND	MAB_r5052	G2659T	NA	*rrl*/23S rRNA, peptidyl transferase	
LZD_r2		1		60	>100	ND	ND	MAB_r5052	G2659T	NA		MAB_3489c
LZD_r3		1		70	>100	ND	ND	MAB_r5052	G2659T	NA		MAB_3489c
LZD_r4		2		70	>100	ND	ND	MAB_r5052	G2659T	NA		
LZD_r5		2		80	>100	ND	ND	MAB_r5052	G2659T	NA		
LZD_r6		1		35	>100	ND	ND	MAB_3820c	T424C	C142R	*rplC*/50S ribosomal protein L3	
LZD_r7		2		30	>100	ND	ND	MAB_3820c	T424C	C142R		
LZD_r8	100	1	2 × 10^−6^	10	40	1.5	15	MAB_4384	27dupC	R9fs	*tetR*, transcriptional repressor of *mmpS5-mmpL5*	
LZD_r9		1		8	40	2	15	MAB_4384	293_294insGAT	Q98QinsM		MAB_0341
LZD_r10		1		12	35	2	15	MAB_4384	27delC	R9fs		MAB_4324c
LZD_r11		2		8	40	2	20	MAB_4384	469_470del	P157fs		MAB_4362, MAB_1072c
LZD_r12		2		15	35	3	15	MAB_4384	217_328del	D73fs		
LZD_r13		2		15	35	3	20	MAB_4384	541dupT	R180fs		
LZD_r14		2		12	40	2.5	20	MAB_4384	200dupT	I67fs		MAB_1681

a WT, wild type; FOR, frequency of resistance (number of resistant colonies per CFU); NA, not applicable; ND, not determined; dup, duplication; ins, insertion; del, deletion; fs, frameshift.

b Independent selection experiments 1 and 2.

### LZD-resistant ribosomal mutations confer strong cross-resistance to other oxazolidinones.

Several other oxazolidinones are in clinical use (tedizolid [TZD]) or in development for the treatment mycobacterial lung diseases (sutezolid [SZD], delpazolid [DZD], and TBI-223), including clinical candidates showing reduced inhibition of mitochondrial protein synthesis compared to LZD, i.e., SZD and TBI-223 (11). To determine whether any of these oxazolidines may overcome LZD resistance, MIC determinations were carried out against representative strains carrying the G2659U mutation in the 23S rRNA (strain LZD_r1) or the C142R amino acid substitution in the 50S ribosomal protein L3 mutation (strain LZD_r6). Mutations in both ribosomal components conferred strong cross-resistance to all oxazolidinones tested (Table 2).

**TABLE 2:** Susceptibility of representative linezolid-resistant M. abscessus ATCC 19977 strains to oxazolidinones and other anti-M. abscessus drugs

Class and drug^*a*^	WT (μM)		LZD_r1 (23S) (μM)		LZD_r6 (L3) (μM)		LZD_r8 (TetR) (μM)		LZD_r8 (+MAB_4384)^*b*^ (μM)		CLR_r1 (23S)^*c*^ (μM)	
	MIC_50_	MIC_90_	MIC_50_	MIC_90_	MIC_50_	MIC_90_	MIC_50_	MIC_90_	MIC_50_	MIC_90_	MIC_50_	MIC_90_
Oxazolidinones												
LZD	3.5	12	>100	>100	30	>100	8	35	5	15	5	15
TZD	0.4	3	70	>100	5	20	1.5	6	1	4.5	2	8
SZD	4	12	>100	>100	40	>100	20	90	5	20	4.5	20
SZD-M1	4	15	>100	>100	40	>100	3	15	4	15	3	15
DZD	2.5	10	100	>100	25	80	2.5	12	3	12	5	20
TBI-223	4	15	>100	>100	60	>100	8	60	5	20	5	25
Other anti-M. abscessus drugs												
CLR	0.3	1.5	0.8	25	0.2	3.0	0.5	2	0.2	2	>100	>100
AZT	3.5	12	12	>100	3	15	3.5	15	2.5	15	>100	>100
AMK	3	12	50	>100	5	20	3.5	12	4	12.5	4	15
TGC	1	3	1.2	25	1	4	1.2	3	1	3	1	5
OMC	2	12	8	50	3	15	4	12.5	4	12	3	12
FOX	10	20	12	25	10	20	10	20	10	20	12	20
BDQ	0.3	0.5	0.4	1	0.3	0.6	0.15	0.4	0.2	0.4	0.3	0.6
CFZ	6	25	50	>100	8	25	4	25	5	25	13	40
RFB	0.3	1	0.5	1	0.5	1	0.5	1	0.5	1.2	0.8	1
MXF	0.5	2.5	1.5	4	1	3	1	3	1	3	2	4
SPR719	0.25	1	0.2	6	0.2	1	1.5	15	0.3	2.5	0.8	1.5

a Drug: LZD, linezolid; TZD, tedizolid; SZD, sutezolid; SZD-M1, sutezolid metabolite; DZD, delpazolid; CLR, clarithromycin; AZT, azithromycin; AMK, amikacin; TGC, tigecycline; OMC, omadacycline; FOX, cefoxitin; BDQ, bedaquiline; CFZ, clofazimine; RFB, rifabutin, MXF, moxifloxacin.

b Strain LZD_r8 carrying a frameshift mutation in the *tetR* gene MAB_4384 (Table 1) complemented with a wild-type copy of MAB_4384 expressed under the control of the *hsp60* promoter as described previously (pMV306-*hsp60*-MAB_4384 [16]). Strain LZD_r8 harboring pMV306-*hsp60* without inserted *tetR* gene did not show any MIC shift.

c CLR-resistant strain M. abscessus ATCC 19977 harboring mutation A2270G in the 23S rRNA (14).

### LZD resistance mutation in 23S rRNA, but not in the ribosomal L3 protein, confers various levels of cross-resistance to several other, nonoxazolidinone anti-M. abscessus antibiotics.

Antibiotic resistance arising via chromosomal mutations typically involves alterations of the binding site of the drugs and is thus specific to a particular class of antibiotics. Recently, Hung and colleagues showed that several mutations in the 23S rRNA of Mycobacterium smegmatis (largely clustered around the peptidyl transferase center [12]), surprisingly altered susceptibility to several structurally and mechanistically unrelated classes of antibiotics (13). Those authors showed that these 23S rRNA mutations increased the generation time and were associated with broad changes of the transcriptome, pointing to pleiotropic consequences of this off-target multidrug resistance mechanism (13). To determine whether the LZD-resistant G2659U mutation in M. abscessus’s 23S rRNA confers cross-resistance, we tested the susceptibility of LZD_r1 to nonoxazolidinone anti-M. abscessus drugs that do and do not target the ribosome. Protein synthesis inhibitors included the macrolides clarithromycin (CLR) and azithromycin (AZT) (binding to the exit tunnel in the 50S ribosomal subunit), the aminoglycoside amikacin (AMK), and the tetracyclines tigecycline (TGC) and omadacycline (OMC) (binding to distinct sites on the 30S ribosomal subunit). Furthermore, the peptidoglycan synthesis inhibitor cefoxitin (FOX, β-lactam), the F-ATP synthase inhibitor bedaquiline (BDQ, diarylquinolone), the redox cycler clofazimine (CFZ, riminophenazine), the RNA synthesis inhibitor rifabutin (RFB, rifamycin), the DNA gyrase poison moxifloxacin (MXF, fluoroquinolone), and the ATPase inhibitor of DNA gyrase SPR719 (benzimidazole) were included. Interestingly, the protein synthesis inhibitors CLR-AZT, AMK, and TGC-OMC, as well as the redox cycler CFZ and the DNA gyrase inhibitor SPR719, showed various levels of cross-resistance (Table 2). This cross-resistance effect was specific to the LZD-resistant G2659U mutation in the 23S rRNA and was not observed for the LZD-resistant C142R mutation in the ribosomal protein L3 (Table 2). We also tested a CLR-resistant M. abscessus ATCC 19977 mutant strain (CLR_r1) (Table 2), isolated on CLR agar (100 μM) after 5 days of incubation, and this strain showed wild-type growth and as it harbored the previously described high-level (MIC > 100 μM) CLR resistance mutation (A2270G; E. coli numbering A2058G) in the 23S rRNA (14) for cross-resistance to the panel of M. abscessus drugs (Table 2). In contrast to the LZD-resistant G2659U mutation, the CLR-resistant A2270G mutation conferred resistance only to CLR (and its macrolide analog AZT) and not to unrelated antibiotic classes (14). This suggested that multidrug resistance conferred by the LZD resistance G2659U mutation is specific and not a general effect of 23S rRNA mutations conferring resistance to inhibitors of protein synthesis. A detailed multiomics analysis may provide insights into the mechanism(s) by which the G2659U 23S rRNA mutation causes decreased susceptibility to multiple diverse antibiotics (14).

### High-frequency and low-level LZD resistance is due to loss-of-function mutations in the transcriptional repressor MAB_4384 that regulates expression of a drug efflux pump.

To explore potential low-level LZD resistance, the single-step mutant selection experiment was repeated with a lower drug concentration (100 μM, 4× the agar MIC), the lowest concentration which suppressed growth of wild-type colonies on agar. Two independent selection experiments revealed a high spontaneous resistance frequency of 2 × 10^−6^/CFU with resistant colonies emerging after 5 days of incubation. Drug-free growth curves of seven resistant colonies (LZD_r8 to _r14) (Table 1) revealed wild-type generation times, suggesting that the resistance mutations do not affect growth. As expected, MIC determinations revealed low-level LZD resistance for strains LZD_r8 to _r14 (Table 1). Whole-genome sequencing, confirmed by targeted Sanger sequencing, identified six distinct frameshift mutations and one amino acid insertion mutation in the TetR transcriptional regulator MAB_4384 (15). A representative frameshift mutant strain (LZD_r8) was complemented with a wild-type copy of MAB_4384 expressed under the control of the *hsp60* promoter as described previously (16), which reverted the resistance phenotype (Table 2), suggesting that the loss-of-function mutations in MAB_4384 are causative of low-level LZD resistance. MAB_4384 is a highly specific transcriptional repressor of the siderophore transporter operon *mmpS5-mmpL5* (MAB_4383c to MAB_4382c) (15). Previous work suggested that this transporter also acts as an efflux pump for thioacetazone analogs (15) and the novel DNA gyrase inhibitor SPR719 (16) in M. abscessus. Loss-of-function mutations in MAB_4384 derepress the *mmpS5-mmpL5* operon, resulting in increased expression of the efflux pump and resistance (15, 17). As expected, all LZD-resistant mutants in MAB_4384 were cross-resistant to SPR719 (Table 1). Our results suggested that low-level LZD resistance is mediated by the MmpL5-MmpS5 efflux pump. In a prior analysis, a possible role of efflux pumps in LZD resistance was investigated by measuring transcript levels of a set of putative pumps (not including Mmpl5-MmplS5) and by applying efflux pump inhibitors (5), indicating the involvement of efflux in LZD resistance in M. abscessus. Whether these additional pumps play a role in LZD resistance awaits genetic analyses.

### LZD-resistant MAB_4384 mutation differentially affects susceptibility to other oxazolidinones.

Due to substrate selectivity, an apparent pump-based resistance mechanism of low-level LZD resistance may differentially affect susceptibility to structurally diverse oxazolidinones. We measured the MICs of a panel of oxazolidinones against the MAB_4384 mutant strain M. abscessus LZD_r8 (Table 2) and found that the activity of different oxazolidinones was indeed differentially affected by mutation in MAB_4384, ranging from low-level cross-resistance for TZD, SZD, and TBI-223 to absence of a detectable effect for DZD. It is noteworthy that the active metabolite of SZD, SZD-M1 (18), was not subject to MAB_4384-mediated resistance, in contrast to the parent molecule. Thus, whereas all tested oxazolidinones showed high-level cross-resistance to ribosomal LZD resistance mutations, some analogs retained their activity against LZD resistance conferred by MAB_4384 mutations, suggesting that these compounds are not substrates of the MmpL5-MmpS5 pump. Whether the differential susceptibility to different oxazolidinones is indeed due to substrate selectivity of the pump awaits bacterial cell pharmacokinetic analyses.

### LZD-resistant MAB_4384 mutation does not affect susceptibility to most other anti-M. abscessus antibiotics.

Previously, it was shown that MAB_4384 mutations cause resistance to thioacetazone analogs and to the DNA gyrase inhibitor SPR719 (16, 17). It was also shown that, in contrast to the MmpL5-MmpS5 homolog of Mycobacterium tuberculosis, the pump is not involved in resistance to BDQ or CFZ in M. abscessus (15, 17). To determine whether the M. abscessus pump is involved in resistance to other anti-M. abscessus antibiotics, we measured the MICs of the nonoxazolidinone drug panel against M. abscessus LZD_r8 (Table 2). This confirmed the lack of resistance to BDQ and CFZ and showed that susceptibility to other anti-M. abscessus drugs was not affected.

### Conclusions.

We have confirmed that high-level LZD resistance is due to on-target ribosomal mutations, located in domain V of the 23S rRNA (G2659U) and the ribosomal protein L3 (C142R), previously identified in other mycobacteria as conferring high-level LZD resistance (7, 9). Both ribosomal mutations conferred high-level resistance to other oxazolidinones in clinical use or in development (Table 3). Interestingly, the LZD resistance mutation G2659U in the 23S rRNA (but not the C142R mutation in the ribosomal protein L3) conferred various levels of cross-resistance to other clinically relevant classes of structurally and mechanistically unrelated protein synthesis inhibitors CLR-AZT, AMK, TGC-OMC, the redox cycler CFZ, and the DNA gyrase inhibitor SPR719 (Table 3). Although the mechanistic basis for this intriguing 23S rRNA-based multidrug resistance remains to be determined, our data support a link between this ancient ribozyme and susceptibility to different classes of antibiotics, previously uncovered by Hung and colleagues (13). We furthermore identified a novel indirect low-level LZD resistance mechanism involving loss-of function mutations in the transcriptional TetR regulator MAB_4384, the repressor of the drug efflux pump MmpL5-MmpS5. Mutations in MAB_4384 conferred low-level cross-resistance to the LZD analogs TZD, SZD, and TBI-223, whereas activity of DZD and the major metabolite of SZD, SZD-M1, was unaffected (Table 3). Mutations in MAB_4384 did not affect activity of clinically used nonoxazolidinone anti-M. abscessus drugs (Table 3). These findings may inform the analysis of clinical resistance to LZD, other oxazolidinones, and other clinically relevant antibiotics.

**TABLE 3:** Summary of linezolid resistance selection, resistance-associated mutated genes, frequencies of resistance, and resistance phenotypes and levels^*a*^

Selection condition	Genes with polymorphism	FOR	Resistance phenotypes and levels
LZD high	23S rRNA	Low	LZD: high
			Other oxazolidinones: high
			Unrelated drugs: low to high to unaffected
	L3 protein	Low	LZD: high
			Other oxazolidinones: high
			Unrelated drugs: unaffected
LZD low	TetR	High	LZD: low
			Other oxazolidinones: low to unaffected
			Unrelated drugs: mostly unaffected

a For details refer to Tables 1 and 2.

## MATERIALS AND METHODS

### Bacterial strain and media.

Mycobacterium abscessus subsp. *abscessus* ATCC 19977 was obtained from the American Type Culture Collection. Liquid cultures were grown in Middlebrook 7H9 broth (catalog number 271310; BD Difco, Sparks, MD, USA) (11). Solid cultures were grown on Middlebrook 7H10 agar (catalog number 262710; BD Difco, Sparks, MD, USA) (11).

### Drug sources.

Drugs were obtained from the following sources: linezolid (MedChemExpress catalog number HY-10394); tedizolid (MedChemExpress catalog number HY-14855); sutezolid (MedChemExpress catalog number HY-10392); sutezolid metabolite SZD-M1 (NIH Reagent Program); delpazolid (MedChemExpress catalog number HY-100180); TBI-223 (MedChemExpress catalog number HY-139398); clarithromycin (Sigma-Aldrich catalog number C9742); azithromycin (MedChemExpress catalog number HY-17506); amikacin (Sigma-Aldrich catalog number PHR1654); tigecycline (Sigma-Aldrich catalog number PZ0021); omadacycline (MedChemExpress catalog number HY-14865); cefoxitin (Sigma-Aldrich catalog number C4786); bedaquiline (MedChemExpress catalog number HY-14881); clofazimine (MedChemExpress catalog number HY-B1046); rifabutin (MedChemExpress catalog number HY-17025), moxifloxacin (Sigma-Aldrich catalog number SML1581); SPR719 (MedChemExpress catalog number HY-12930). All drugs were dissolved in dimethyl sulfoxide at a 10 mM concentration except amikacin, which was dissolved in water.

### MIC determinations.

MICs were determined employing the broth microdilution method using 96-well titer plates as described elsewhere (11). Dose-response curves were established using the OD_600_ as readout (Tecan Infinite Pro 200 plate reader). From the dose-response curves, MIC_50_ and MIC_90_, the concentrations that inhibited growth by 50 or 90% compared to the untreated control, were derived (11). All MIC determinations in this work were carried out at least twice, yielding similar results, and mean values are shown.

### Selection of resistant mutants.

Linezolid-resistant strains were isolated on Middlebrook 7H10 agar as described previously (19) containing either 100 μM drug (the lowest concentration suppressing growth of wild-type colonies) or 400 μM for the isolation of high-level resistant mutants. Frequencies of spontaneous resistance (resistant CFU divided by plated CFU) were determined as described previously (20).

### Whole-genome and Sanger sequencing.

Whole-genome sequencing and bioinformatics analyses were carried out by Novogene Corporation Inc. as described previously (19). Sanger sequencing was carried out by Genewiz, Inc., South Plainfield, NJ, USA, as described elsewhere (20). The GenBank accession number for the sequence of the parent strain M. abscessus ATCC 19977 is CU458896.1.

### Genetic complementation.

The M. abscessus ATCC 19977 strain LZD_r8, carrying a frameshift mutation in the *tetR* gene MAB_4384 (Table 1), was complemented with a wild-type copy of MAB_4384 expressed under the control of the *hsp60* promoter as described previously (pMV306-*hsp60*-MAB_4384) (16).

### Data availability.

Sequencing data are available from the authors upon request.

## Supplementary Material

Reviewer comments
